# When to use a tourniquet in primary total knee arthroplasty? A systematic review and meta-analysis

**DOI:** 10.3389/fsurg.2026.1720765

**Published:** 2026-03-19

**Authors:** Shujiang Wang, Xu Shen, Beiyue Wang

**Affiliations:** 1 Department of Outpatient, Jinling Hospital affiliated of Nanjing University, Nanjing, China; 2 Department of Joint Surgery, The Second Affiliated Hospital of Nanjing Medical University, Nanjing, China

**Keywords:** cementation, duration, surgery, TKA, tourniquet

## Abstract

**Purpose:**

This study aimed to investigate the optimal timing strategy for tourniquet use in primary total knee arthroplasty (TKA).

**Methods:**

A systematic search of PubMed, EMBASE, Web of Science, and the China National Knowledge Infrastructure was conducted to identify randomized controlled trials published up to January 2023. The included studies compared the use of a tourniquet solely during cementation (specific-duration tourniquet, SDT) vs. its use throughout the majority of the surgical procedure (majority-duration tourniquet, MDT). MDT was defined as tourniquet inflation prior to the surgical incision and deflation only after the cement had hardened. Continuous variables were pooled using the weighted mean difference (WMD), and relevant subgroups were analyzed independently.

**Results:**

Six studies involving 358 patients (386 knees) were included and assigned to either the SDT or MDT group. Meta-analysis showed that while the SDT group experienced higher intraoperative blood loss [WMD = −68.62, 95% confidence interval (CI): (−93.72 to −43.52), *P* < 0.001], there was no increase in total blood loss (*P* = 0.82). The SDT technique did not increase operative time (*P* = 0.16), yet it improved the postoperative knee visual analog scale scores [WMD = 0.77, 95% CI: (0.31–1.23), *P* = 0.001] and knee range of motion (ROM) 3 days postoperatively [WMD = −6.69, 95% CI: (−9.29 to −4.08), *P* < 0.00001]. Meanwhile, no significant difference in ROM was observed between the groups 2 weeks postoperatively (*P* = 0.31). Finally, the SDT group was associated with a decreased risk of postoperative complications [risk ratio2.77; 95% CI: (1.04–7.43); *P* = 0.04].

**Conclusion:**

The SDT strategy may be associated with a reduced risk of postoperative complications and accelerated early functional recovery compared to the MDT strategy in primary TKA. Therefore, tourniquet application during SDT appears to be the optimal timing for primary TKA.

## Background

Primary total knee arthroplasty (TKA) is the treatment for various causes of knee pain in patients with end-stage osteoarthritis (1). The use of a tourniquet is standard practice in primary TKA, owing to its efficacy in minimizing intraoperative hemorrhage, ensuring a clear operative field, and reducing overall procedure times (2, 3). However, tourniquet use is associated with a variety of complications, including limb swelling, muscle injury, poor wound healing, wound infection, nerve palsy, deep vein thrombosis (DVT), and delayed postoperative functional recovery (4–6). At present, some studies have investigated the effects of tourniquet release at different intervals on blood loss and postoperative complications in primary TKA (7–9). Four main tourniquet application strategies are recognized in primary TKA: (1) whole-duration tourniquet (WDT), used from skin incision to wound closure; (2) specific-duration tourniquet (SDT), applied only during implant cementation; (3) majority-duration tourniquet (MDT), inflated before the incision and deflated after cement hardening; and (4) posterior majority-duration tourniquet (PMDT), inflated before cementation and deflated after wound closure. Wang et al. (10) demonstrated that tourniquet use in SDT was associated with a decreased risk of postoperative complications and faster functional recovery in primary TKA. However, this strategy did not effectively limit intraoperative or total blood loss compared to the WDT technique. Kvederas et al. concluded that tourniquet use in MDT increased blood loss but decreased the risk of early postoperative complications compared with the WDT group (11). Cao et al. demonstrated that tourniquet use in PMDT significantly reduced hidden blood loss, lowered the incidence of postoperative complications, and relieved tissue swelling (12). Nevertheless, the optimal timing for tourniquet use remains controversial. To our knowledge, no previous trials have directly compared the SDT and MDT strategies in primary TKA. Therefore, we conducted a meta-analysis to compare these two tourniquet application strategies to identify the optimal tourniquet timing for primary TKA.

## Methods

This systematic review was conducted in accordance with the PRISMA guidelines. Two reviewers independently searched the Medline, Embase, Cochrane Library, Web of Science, and China National Knowledge Infrastructure (CNKI) databases for studies published up to January 2023. In this study, the following keywords were utilized: ((total knee arthroplasty) OR (TKA)) AND ((tourniquet) OR (tourniquet less)). A manual search of the reference lists of all identified publications was performed to retrieve any additional potentially eligible studies.

### Inclusion and exclusion criteria

Studies were selected for inclusion according to the patient, intervention, comparison, outcome, study design (PICOS) criteria: (1) patients undergoing primary TKA; (2) randomized controlled trials (RCTs); and (3) two tourniquet protocols were compared: one with inflation limited to the cementation period vs. another with inflation maintained from skin incision until cement hardening. Exclusion criteria were as follows: (1) revision TKA or complicated TKA, (2) non-randomized controlled trials, (3) animal studies, and (4) the use of cementless and hybrid prosthesis for implanting.

### Data extraction

Two review authors independently extracted data using a pre-designed, standardized data collection form. Any discrepancies were resolved through discussion or by consultation with the corresponding author. Relevant data included publication information, the authors, study design, patient basic information, tourniquet duration, operative time, intraoperative blood loss, total blood loss, knee pain scores (visual analog scale, VAS), knee range of motion (ROM), and postoperative complications.

### Quality assessment

The methodological quality of the included studies was independently assessed by two researchers in accordance with the Cochrane Handbook's recommended criteria. The risk of bias was evaluated across seven domains: random sequence generation, allocation concealment, blinding of participants and personnel, blinding of outcome assessment, incomplete outcome data, selective reporting, and other potential biases. For each domain, the risk of bias was judged to be “low,” “unclear,” or “high” based on the information available in the studies.

### Statistical analysis

The systematic review was performed using Review Manager (RevMan) version 5.3. Data were analyzed by calculating weighted mean differences (WMDs) for continuous variables and risk ratios (RRs) for dichotomous variables, both with 95% confidence intervals (CIs). Statistical significance was set at *p* <0.05. The degree of statistical heterogeneity was evaluated using the chi-square test and the *I*^2^ statistic. Based on pre-defined criteria, *I*^2^ values were categorized as low (<25%), moderate (25%–50%), or high (>75%).

## Results

### Study selection

**Figure 1:**
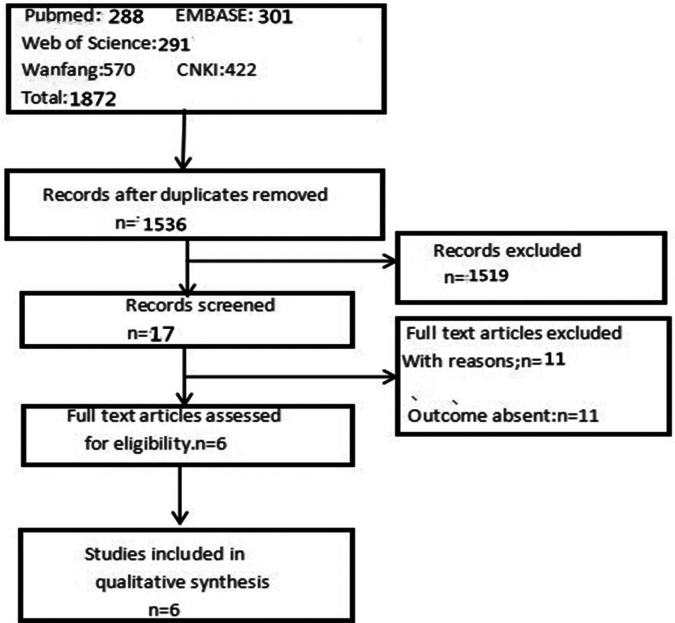
Flowchart of study search and selection.

A total of 1,872 relevant articles were retrieved through the initial database search. According to the inclusion criteria, 1,536 articles were remained after removing 336 duplicates; based on a review of titles and abstracts, 17 articles were retained. Then, we downloaded these articles; of these, 11 were excluded for various reasons. Finally, six studies (11, 13–17) met the eligibility criteria and were included in the meta-analysis (Figure 1).

### Study characteristics and quality assessment

**Figure 2:**
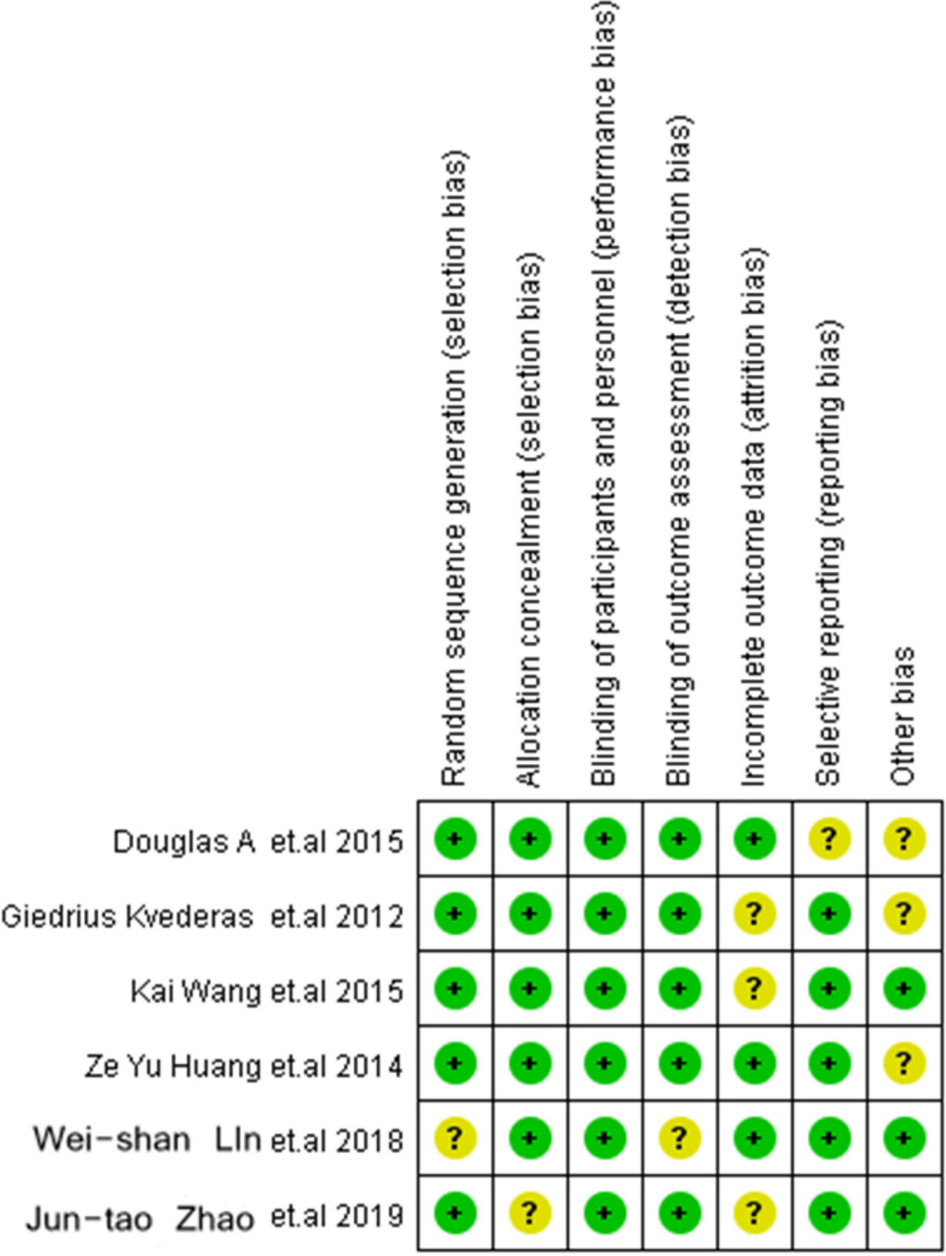
Summary of bias risk.

The six included studies involved a total of 358 patients and 386 knee arthroplasties. There were 201 arthroplasties in the MDT group and 185 in the SDT group. Their baseline characteristics are presented in Table 1, and the results of the quality assessment are summarized in Figure 2.

**Table 1:** Characteristics of studies.

Study	Knees		Total	Gender (M/F)		Mean age (years)		BMI (kg/m^2^)		Results
	MDT	SDT		MDT	SDT	MDT	SDT	MDT	SDT	
Wang et al. (2015)	25	25	50	5/20	4/21	72.3 ± 7.1	72.5 ± 6.8	28.8 ± 6.2	29.1 ± 6.6	1/2/3/4/5/6/7/8
Huang et al. (2014)	30	30	60	10/20	11/19	66.1 ± 5.8	66.3 ± 6.1	25.9 ± 3.3	26.5 ± 2.4	1/2/4/5/7/8
Kvederas et al. (2012)	12	12	24	1/11	3/9	68.2 ± 6.4	67.3 ± 6.4	32.0 ± 2.8	31.1 ± 4.8	1/2
Douglas et al. (2015)	14/28	14/28	28/56	—	—	—	—	—	—	1/2/3/4/5/6/7
Lin et al. (2018)	50	50	100	25/25	26/24	60.3 ± 8.6	60.2 ± 8.4	—	—	1/2/3/4/8
Zhao et al. (2019)	56	40	106	16/40	11/29	67.23 ± 7.56	68.33 ± 9.26	25.86 ± 3.86	24.76 ± 4.46	1/2/3/4/6/8

1, tourniquet duration; 2, operative time; 3, intraoperative blood loss; 4, total blood loss; 5, knee VAS score on 3 days postoperatively; 6, knee ROM on 3 days postoperatively; 7, knee ROM on 3 days postoperatively; and 8, complications.

### Blood loss

**Figure 3:**
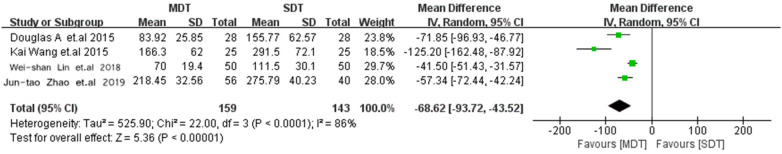
Forest plot comparing intraoperative blood loss between the MDT and SDT groups.

**Figure 4:**
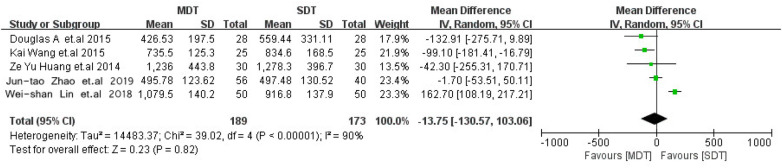
Forest plot comparing total blood loss between the MDT and SDT groups.

Four studies (14–17), including 159 patients in the MDT group and 143 patients in the SDT group, reported intraoperative blood loss (IBL) and demonstrated significantly higher blood loss in the SDT group (WMD, −68.62; 95% CI, −93.72 to −43.52; *p* < 0.001) (Figure 3). Five studies (13–17), including 189 patients in the MDT group and 173 patients in the SDT group, reported total blood loss (TBL); however, no significant difference was observed between the two groups (WMD, −13.75; 95% CI, −30.57 to 103.06; *p* = 0.82; Figure 4).

### Operative time

**Figure 5:**
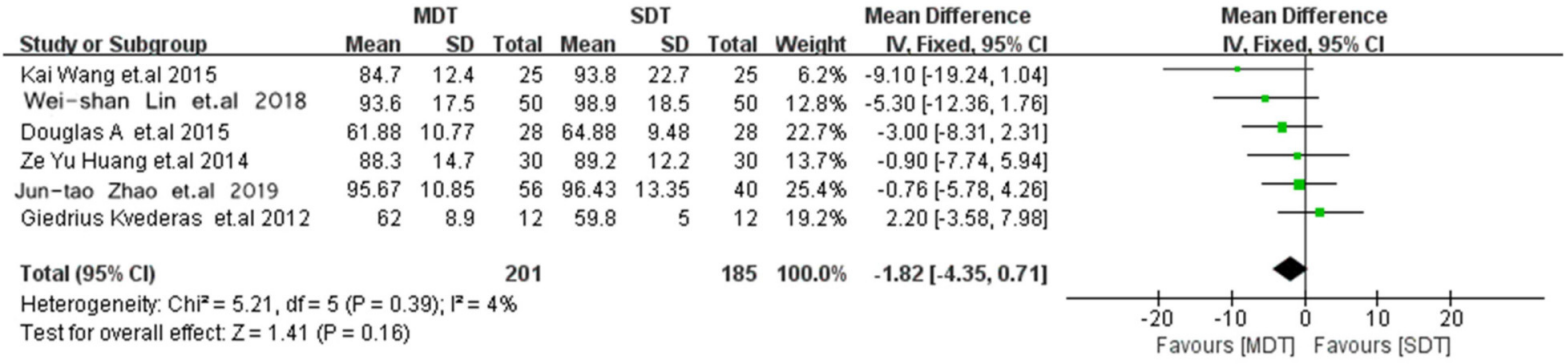
Forest plot comparing operative time between the MDT and SDT groups.

Six studies (11, 13–17) reported operative time during TKA and demonstrated no significant difference between the MDT and SDT groups (MD = −1.82; 95% CI −4.35 to 0.71; *p* = 0.16; Figure 5).

### Tourniquet duration

**Figure 6:**
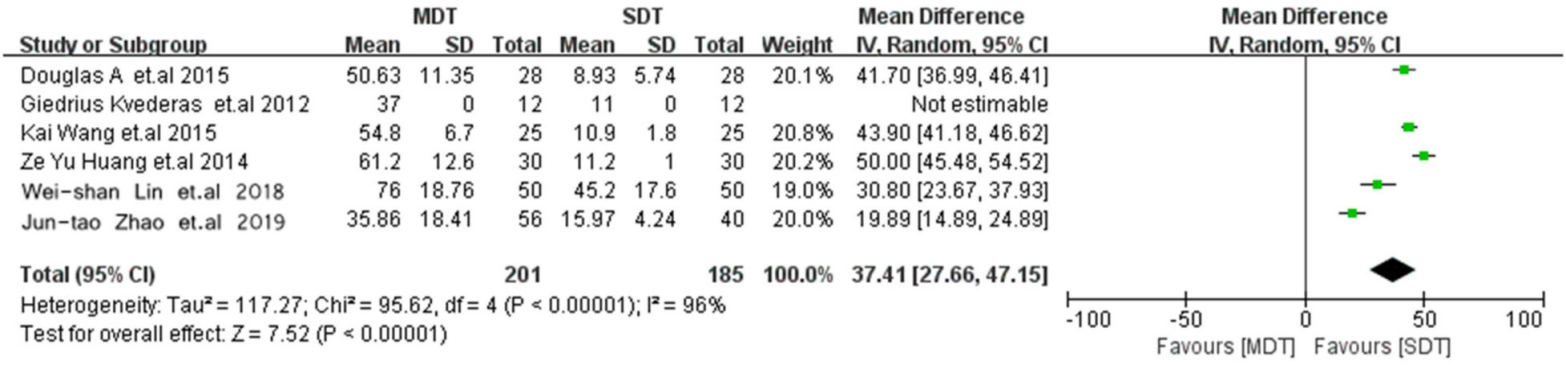
Forest plot comparing tourniquet duration between the MDT and SDT groups.

Across six studies, a significant reduction in tourniquet duration was observed in the SDT group (MD = 37.41; 95% CI 27.66–47.15; *p* < 0.00001; Figure 6) (11, 13–17).

### Functional assessment

**Figure 7:**
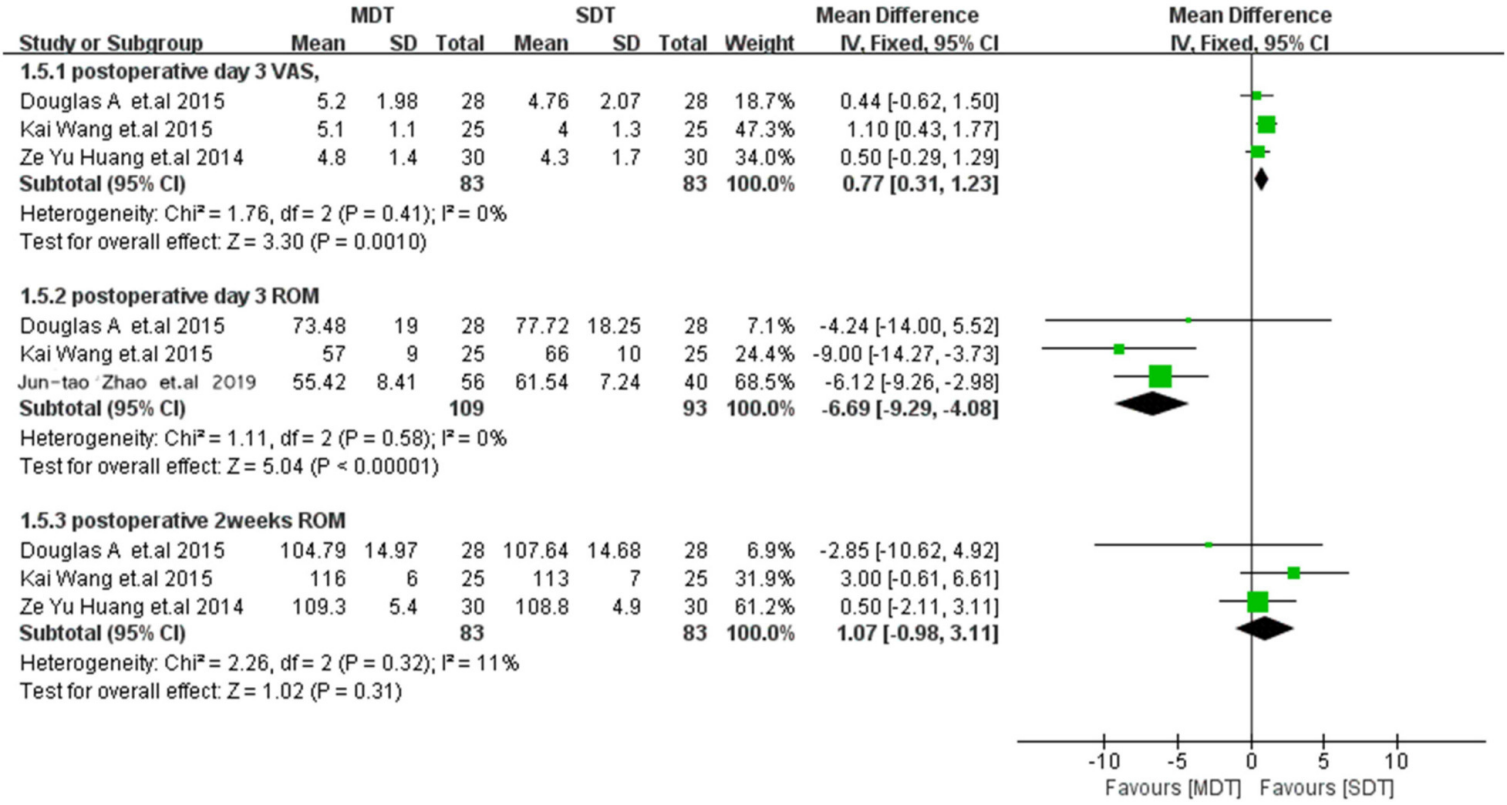
Forest plot comparing functional assessment between the MDT and SDT groups.

Three studies (13–15) reported VAS scores for knee pain on postoperative day 3 (POD 3), showing significantly lower knee pain scores in the SDT group (WMD = 0.77; 95% CI 0.31–1.23; *p* = 0.001). Three studies (14, 15, 17) reported active knee ROM on POD 3 and showed a significantly increased ROM in the SDT group (WMD = −6.69; 95% CI −9.29 to −4.08; *p* < 0.00001). Three studies (13–15) examining ROM at postoperative 2 weeks (PO 2W) showed no significant difference between the two groups (WMD = 1.07; 95% CI −0.98 to 3.11; *p* = 0.31; Figure 7).

### Complications

**Figure 8:**
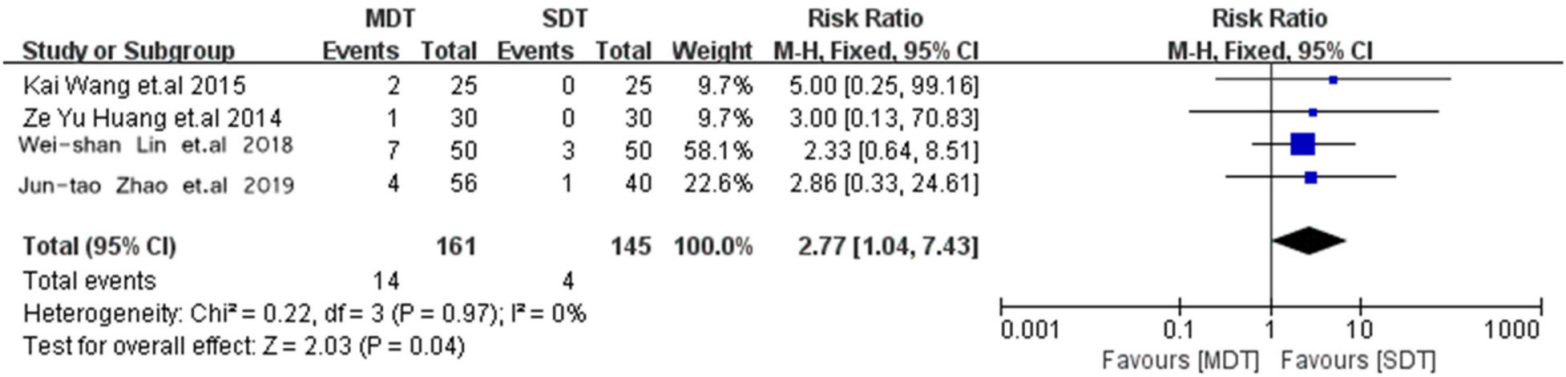
Forest plot comparing postoperative complications between the MDT and SDT groups.

Four studies (13, 15–17) reported postoperative complications, including DVT, wound infection, nerve palsy, and hematoma. We demonstrated that the SDT group had a significantly lower risk of complications compared to the MDT group (RR = 2.77; 95% CI 1.07–7.43; *p* = 0.04; Figure 8). Because of fewer cases of DVT, wound infection, nerve palsy, and hematoma, we could not perform subgroup analyses in this meta-analysis.

## Discussion

At present, although tourniquets are widely used in primary TKA, the optimal timing for their release remains controversial (18, 19). So far, no meta-analysis has evaluated the effects of the SDT and MDT tourniquet application strategies. To our knowledge, this study represents the first meta-analysis to compare the efficacy of the SDT and MDT tourniquet techniques. The primary conclusion of this meta-analysis is that despite being associated with higher intraoperative blood loss, the SDT technique reduces the risk of postoperative complications and enhances early functional recovery following TKA.

Regarding blood loss, the results demonstrated that the MDT group significantly reduced intraoperative blood loss and was not associated with total blood loss. Therefore, we conclude that the MDT group may lead to increased postoperative hidden blood loss. This phenomenon likely occurs because the tourniquet increases fibrinolytic activity, which promotes bleeding into the joint and surrounding tissues after the procedure, which is consistent with the results reported by Douglas et al. (7).

Regarding operative time, the duration mainly depends on the surgical technique of the surgeons in primary TKA. The tourniquet application during primary TKA was believed to effectively provide a relative bloodless surgical field (20). In this article, the operative time of the MDT group decreased by only 1.81 min compared with the SDT group, which had no clinical significance. According to the report of Nicolaiciuc et al., the absence of a tourniquet does not adversely affect surgical field visibility, a conclusion supported by our data (21).

Postoperative functional recovery is particularly important for primary TKA (22). Forest plot analysis revealed that tourniquet use during SDT significantly improved early outcomes (lower VAS pain scores and increased ROM on POD3), indicating that tourniquet-free TKA may impede early rehabilitation. This may be due to direct damage to the tourniquet, and reperfusion injury might increase pain. Similar results were reported by Zhang et al. (23). Meanwhile, Liu et al. (24) attributed limb swelling in the MDT group to hidden blood loss escaping into the joint space and surrounding soft tissue. Such swelling requires greater muscle strength to perform functional training but there was no significant difference at 2 weeks after surgery.

Regarding complications, this study found that the SDT technique significantly reduced the risk of complications. Because there are fewer cases of DVT, wound infection, nerve palsy, and hematoma, we could not perform subgroup analyses in this meta-analysis. Previous studies have identified wound infection and thromboembolism as the most common complications after TKA (24, 25). Prolonged tourniquet application may impede blood flow and prevent antibiotics from reaching the surgical wound, which can lead to excessive inflammation, muscle damage, and an increased risk of wound infection (26–28). Similarly, two previous meta-analyses have found no significant association between tourniquet use during primary TKA and the occurrence of DVT or PE (29, 30). Since the duration of tourniquet application remained under 100 min in all cases, we infer that there would be no significant differences between the groups. Nevertheless, this hypothesis needs more studies for validation.

This meta-analysis has several limitations. First, the inclusion of only six RCTs may affect the precision of the outcomes. Second, some complicated factors such as varying tourniquet pressures, different types of intraoperative prostheses, and different methods for measuring blood loss may lead to certain heterogeneity. Third, the low incidence of DVT, wound infection, nerve palsy, and hematoma precluded the use of subgroup analyses to further explore the complications associated with tourniquet use. The potential influence of tourniquet on prosthesis loosening quality has not yet been evaluated; therefore, longer follow-up periods are needed to monitor the complication of prosthesis loosening. In addition, future studies should employ sample size calculations based on the minimal clinically important differences (MCID) and report the proportion of patients who achieve the MCID (responder analysis) to provide more clinically meaningful evidence. Consequently, further high-quality randomized controlled trials are required to comprehensively assess the tradeoffs of various tourniquet strategies in primary TKA.

## Conclusion

The application of a tourniquet solely during cementation (SDT) not only lowers the risk of postoperative complications but also facilitates faster functional recovery in the early phase after TKA. Therefore, tourniquet application in SDT represents the optimal timing during primary TKA.

## Data Availability

The original contributions presented in the study are included in the article/Supplementary Material; further inquiries can be directed to the corresponding author.
